# Temperature alters *Plasmodium* blocking by *Wolbachia*

**DOI:** 10.1038/srep03932

**Published:** 2014-02-03

**Authors:** Courtney C. Murdock, Simon Blanford, Grant L. Hughes, Jason L. Rasgon, Matthew B. Thomas

**Affiliations:** 1 Center for Infectious Disease Dynamics and Department of Entomology, Pennsylvania State University, University Park, PA, USA

## Abstract

Very recently, the Asian malaria vector (*Anopheles stephensi*) was stably transinfected with the *w*AlbB strain of *Wolbachia*, inducing refractoriness to the human malaria parasite *Plasmodium falciparum*. However, conditions in the field can differ substantially from those in the laboratory. We use the rodent malaria *P. yoelii*, and somatically transinfected *An. stephensi* as a model system to investigate whether the transmission blocking potential of *w*AlbB is likely to be robust across different thermal environments. *w*AlbB reduced malaria parasite prevalence and oocyst intensity at 28°C. At 24°C there was no effect on prevalence but a marked increase in oocyst intensity. At 20°C, *w*AlbB had no effect on prevalence or intensity. Additionally, we identified a novel effect of *w*AlbB that resulted in reduced sporozoite development across temperatures, counterbalancing the oocyst enhancement at 24°C. Our results demonstrate complex effects of temperature on the *Wolbachia*-malaria interaction, and suggest the impacts of transinfection might vary across diverse environments.

Despite intensive control efforts, malaria remains one of the most important infectious diseases worldwide^1^. Manipulation of mosquitoes to render them ineffective at transmitting parasites and pathogens could create exciting new opportunities for control of vector-borne diseases e.g.^2^,^3^,^4^,^5^,^6^. One of the most promising approaches involves transinfection of mosquitoes with strains of the maternally inherited endosymbiotic bacteria, *Wolbachia* (reviewed in^7^,^8^,^9^,^10^,^11^,^12^). Studies with a range of mosquito and parasite/pathogen species have shown *Wolbachia* to reduce vector competence and/or vectorial capacity^4^,^13^,^14^,^15^,^16^,^17^,^18^,^19^,^20^,^21^,^22^,^23^,^24^,^25^,^26^. Further, *Wolbachia* possesses a genetic drive mechanism (cytoplasmic incompatibility) that enhances its own spread through host populations by skewing normal Mendelian inheritance ratios^27^. Research on the use of *Wolbachia* for control of dengue has progressed to the level of controlled field releases of transinfected *Aedes aegypti* mosquitoes^8^,^28^. Research on malaria mosquito vectors has proven more challenging, but a stably transinfected line of *Anopheles stephensi* has recently been developed and evaluated in the laboratory^4^.

The mechanisms underlying the transmission blocking properties of *Wolbachia* remain unclear, but appear linked to aspects of mosquito immune function^4^,^15^,^19^,^20^,^21^,^29^,^30^,^31^, and possible competition for resources within the mosquito^15^. Recent research has demonstrated that mosquito immune function can vary considerably with modest changes in temperature^32^,^33^,^34^. It is also well established that growth of malaria parasites within the mosquito is strongly temperature-dependent^35^. Furthermore, studies from a wide diversity of non-vector systems illustrate that *Wolbachia* replication, dissemination, vertical transmission, fitness effects and the extent of cytoplasmic incompatibility can all vary with temperature (SI Table 1). This range of thermal sensitivities raises the possibility that patterns of *Wolbachia*-induced transmission blocking might depend strongly on the local environment. If so, insights gained under standard insectary conditions at 27°C may reveal little about the natural environments for malaria transmission where mean temperatures can range from 18 to 34°C, and daily temperature variation frequently exceeds 10°C^36^,^37^,^38^. For example, blocking could be enhanced under certain conditions, increasing the effectiveness of the approach. Alternatively, variation in temperature might reduce blocking. The worst-case scenario is that malaria infection might actually be enhanced in certain environments by the interaction between *Wolbachia* and *Plasmodium*^39^.

As proof of principle, we use a rodent malaria *P. yoelii* and somatically transinfected *An. stephensi* as a model to investigate how changes in temperature influence the *Wolbachia*-malaria parasite interaction. If temperature significantly influences pathogen blocking, such effects would suggest that the impacts of transinfection cannot be determined from studies conducted under one set of conditions alone.

## Results

### Effects of temperature on *Wolbachia* density and mosquito survival

**Figure 1:**
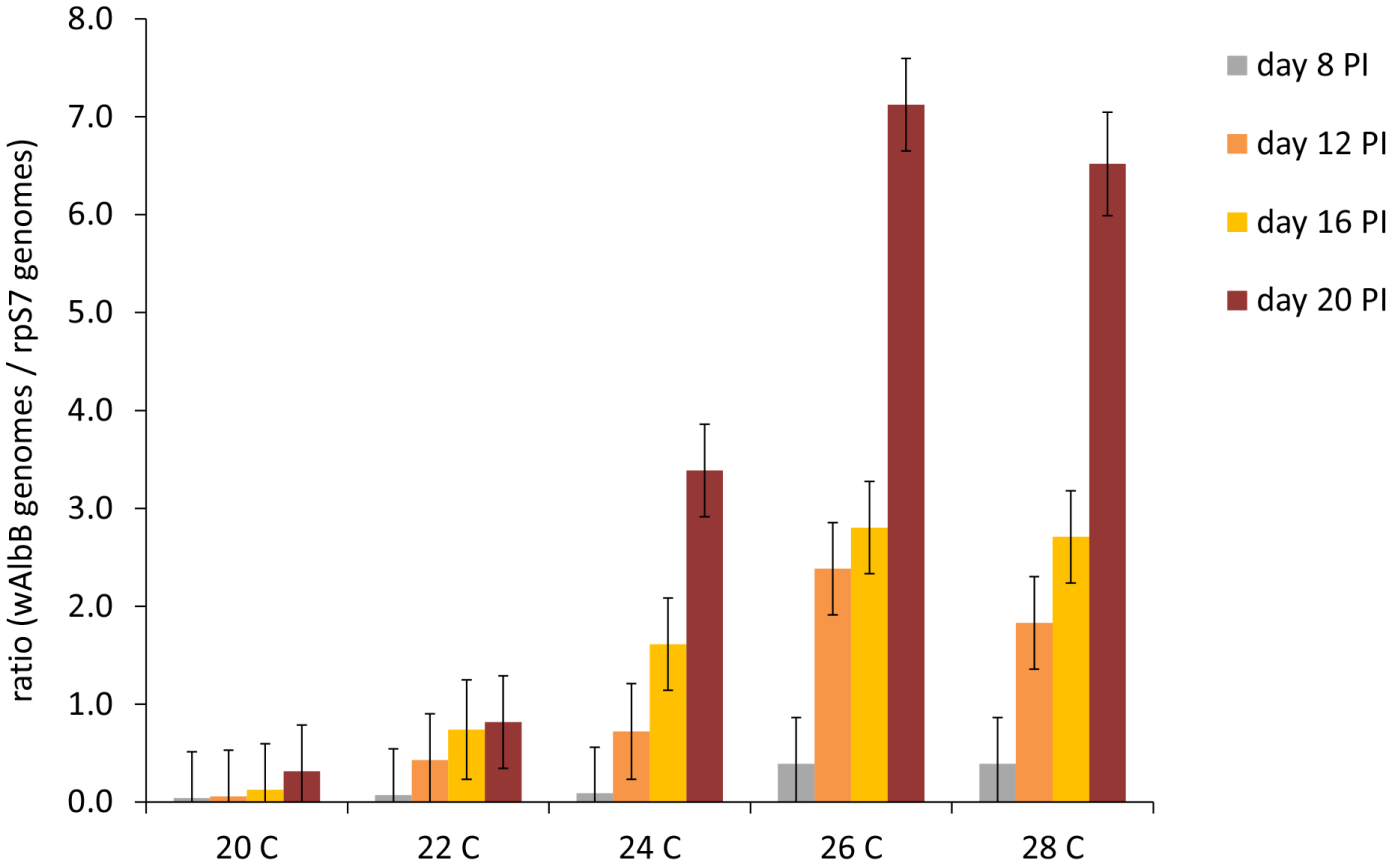
Temperature, sampling time point, and their interaction significantly influenced *w*AlbB replication in whole mosquito carcasses. *w*AlbB density (ratio of *w*AlbB to host *rpS7* genomes) is clearly mediated by temperature, with the rate of replication significantly increasing in mosquitoes housed at 26°C compared to those housed at 24°C, and no significant changes through time in *w*AlbB densities occurring in mosquitoes housed at 20°C and 22°C. Bars around mean values represent standard errors.

The density of *Wolbachia* increased over time (Fig. 1, Table 1), with mosquitoes sampled on day 20 exhibiting significantly higher densities of *w*AlbB than on days 16 (p < 0.0001), 12 (p < 0.0001), and 8 (p < 0.0001) post-injection. *Wolbachia* densities were also significantly higher in the warmer temperatures of 26°C and 28°C, compared with cooler temperatures (20°C vs. warmer temperatures, p < 0.0001; 22°C vs. 24°C, p = 0.017; 22°C vs. 26°C and 28°C, p < 0.0001; 24°C vs. warmer temperatures, p < 0.0001; Table 1). A significant interaction between sampling day and temperature (*days post-Wolbachia infection x temperature*) indicated that the rates of *w*AlbB replication increased with warming temperatures, with no significant increases in *w*AlbB densities occurring at 20°C and 22°C (Fig. 1).

There was no significant effect of temperature or *Wolbachia* infection on mosquito survival (Supplementary Information Text S2). There was a minor transient effect of micro-injection on mortality within the first 24-48 hrs, and this was consistent between the *w*AlbB and Sua5B control treatments (Supplementary Information Text S2 Fig. 1; Sua5B cell control: *X*^2^ = 106.28, p < 0.0001); *w*AlbB: *X*^2^ = 113.48, p < 0.0001). After the first two days of the experiment, the Sua5B control and *w*AlbB survival curves resemble that of the unmanipulated population (Supplementary Information Text S2).

### Effects of temperature on *Plasmodium* blocking

**Figure 2:**
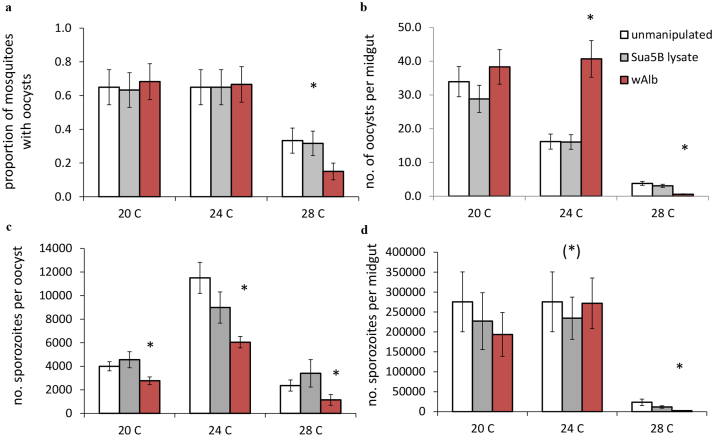
Temperature shaped the *w*AlbB-malaria interaction in complex ways. (a) Temperature alone significantly affected oocyst prevalence (the proportion of mosquitoes with oocysts on their midguts), with significant declines in oocyst prevalence occurring at 28°C for all treatment groups (asterisk represents significant pairwise comparisons for each treatment group at 28°C with treatment groups placed at lower temperatures, p < 0.05). (b) Temperature significantly mediated the effect of *w*AlbB on oocyst intensity (the number of oocysts per midgut). *w*AlbB infection either enhanced (24°C), blocked (28°C), or had no effect on the number of establishing oocysts (20°C). (c) Infection with *w*AlbB significantly reduced the number of sporozoites produced per oocyst across all temperature treatments. (d) Temperature significantly mediated the effect to *w*AlbB on the total number of sporozoites produced per mosquito, with significant declines in overall sporozoite production at 28°C in *w*AlbB infected mosquitoes. Bars represent the unadjusted means of each response variable, while whiskers portray the standard error around the mean. Asterisks in (b–d) denote significant pair-wise comparisons within a temperature between *w*AlbB and the other treatment groups. The asterisk within parentheses at 24°C indicates a significant effect of *w*AlbB infection in explaining variation between the estimated marginal means in the full statistical model, but there is no significant effect when the unadjusted treatment means are compared.

There was an effect of temperature on the probability of a mosquito becoming infected with *P. yoelii* (Table 1, Fig. 2a), with oocyst prevalence being significantly lower at 28°C (p < 0.0001) compared with cooler temperatures. There was no additional effect of *Wolbachia* on parasite prevalence. However, temperature and *Wolbachia* did interact to impact oocyst intensity (Table 1, Fig. 2b). At 20°C, the number of oocysts per mosquito midgut did not differ between treatments. At 24°C, infection with *w*AlbB significantly facilitated the establishment of oocysts, such that the transinfected mosquitoes had more than double the oocyst burdens of the Sua5B control and unmanipulated control mosquitoes (Sua5B lysate, p < 0.0001; unmanipulated, p = 0.01). At 28°C on the other hand, infection with *w*AlbB limited the number of establishing oocysts, reducing oocyst burdens by approximately 80% relative to control groups (Sua5B lysate, p < 0.0001; unmanipulated, p < 0.0001).

We next examined the number of sporozoites per oocyst to provide a measure of parasite replication rate. Infection with *w*AlbB significantly reduced the number of sporozoites produced per oocyst across all temperatures, irrespective of *Wolbachia* infection status (Table 2, Fig. 2c). In general there was a relationship between the number of oocysts per midgut (oocyst intensity) and the number of sporozoites produced per oocyst. This negative effect of oocyst density was most marked in the transinfected mosquitoes and temperatures sub optimal for parasite development (20°C and 28°C). This was especially the case for *w*AlbB infected mosquitoes at 28°C (Regression on model residuals: *B* = −0.203, R^2^ = 0.867, F_1,8_ = 21.111, p = 0.002), as illustrated by a significant interaction between treatment and oocyst intensity observed at 28°C (Table 2).

Finally, we used total sporozoites per mosquito midgut as a measure of overall infection intensity (and hence ultimate transmission potential). With this overall measure, GZLM model analyses predicted that infection with *w*AlbB significantly reduced the total number of sporozoites produced at 24°C (unmanipulated vs. *w*AlbB, p < 0.0001; Sua5B lysate vs. *w*AlbB, p < 0.0001) and 28°C (unmanipulated vs. *w*AlbB, p < 0.0001; Sua5B lysate vs. *w*AlbB, p = 0.002; Table 2) relative to the controls and no significant effect of *Wolbachia* on malaria infection at 20°C (Table 2). However, when comparing the unadjusted means at 24°C to model estimates, we did not observe a significant difference in total sporozoite production in the *w*AlbB treatment group relative to the controls (Fig. 2d). This is most likely because the majority of the variance in the model is explained by the positive relationship between oocyst intensity and sporozoites produced per midgut. Thus, while the contribution of treatment in the model is small, it still significantly predicts the remaining variation unexplained by our covariate, with *w*AlbB infected mosquitoes producing fewer sporozoites (Supplementary Information Text S2). This result indicates that enhancement of oocyst intensity by *w*AlbB at 24°C was more or less counterbalanced by the negative effects *w*AlbB infection on parasite replication rate.

At 28°C *w*AlbB caused significant reductions in overall infection intensity since both numbers of oocysts and sporozoite replication were negatively affected (Sua5B cells, p = 0.003; unmanipulated, p = 0.010; Table 2, Fig. 2d). We did see replicate effects at one temperature for both the sporozoite per oocyst and sporozoite per midgut analyses, likely due to variation in infection intensities between individual mice (Table 2). However, there were no significant interactions between replicate and temperature or treatment, so the replicate effects appear to have little influence on the results overall.

## Discussion

Here we use a rodent malaria and somatically transinfected *An. stephensi* as a model to investigate for the first time how changes in temperature influence the *Wolbachia*-malaria parasite interaction. We show that temperature significantly affects *Wolbachia* replication in the mosquito vector and alters the extent and apparent mode of action of transmission blocking. Temperature affected the replication kinetics of *Wolbachia,* establishment and replication of the malaria parasite, and the *Wolbachia*-parasite interaction. Infection with *w*AlbB reduced oocyst intensity at 28°C, increased oocyst intensity at 24°C and had no effect at 20°C. Oocyst intensity is a common measure used to estimate parasite transmission blocking^21^,^39^. In this context, our results demonstrate that somatic infection with *w*AlbB can partly block, enhance, or have no impact on infection depending on temperature. However, *w*AlbB also appears to interfere with parasite replication, reducing the number of sporozoites produced per oocyst. When these effects are combined, *w*AlbB reduces transmission potential strongly at 28°C but has no overall effect at 20°C, or the thermal optimum for *P. yoelii* development, 24°C. The potential for such marked temperature-dependence in transmission blocking phenotypes has not been considered previously.

Use of a rodent malaria complicates direct extension of our results to human malaria. Successful sporozoite invasion of the salivary glands by *P. yoelii* is inconsistent, especially at 28°C^35^, which is why we used sporozoite load within the midgut as our measure of overall infection intensity. Quantification of sporozoite intensity within the salivary glands, as is more reliable with *P. falciparum*, would provide a more definitive measure of pathogen blocking. We also used somatically infected mosquitoes rather than the recently developed stable transinfected line. While there has been little research to date comparing somatic infection and stable transinfection with *Wolbachia*, previous studies indicate that apart from the ovarian tissues, the density and distribution of *Wolbachia* within host tissues is similar between somatic and stable infections, both infection strategies impair development of *P. falciparum* at the oocyst stage^4^,^21^, and modulate similar expression levels of immune genes^4^,^20^,^21^,^29^,^31^. Further, the establishment and development rate of both human and rodent malarias are temperature sensitive, even though the absolute thermal performance profiles differ between species. Similar to earlier work investigating the effects of temperature on *P. yoelii*^35^, we found negative effects of temperature on *P. yoelii* oocyst establishment in the midgut at 28°C. We would not expect *P. falciparum* oocyst establishment to be negatively affected until temperatures exceed 30°C^40^. However, this is not an unusual temperature for *An. stephensi* to experience in the field^37^. Moreover, *P. vivax*, which is the other key species of human malaria transmitted by *An. stephensi*, has a lower temperature threshold than *P. falciparum*^37^. Accordingly, there is little reason to believe the influence of temperature to be unique to our model system.

We found replication of *w*AlbB to be temperature sensitive, with an apparent optimum between 26°C and 28°C. Studies in numerous systems show *Wolbachia* replication to increase towards some thermal optimum and then decline as temperatures increase further (Supplementary Information Table S1). Consistent with previous research conducted on *An. gambiae*, we found no effect of somatic infection on mosquito mortality, irrespective of whether or not a mosquito received a bloodmeal^21^. At 28°C, we found a reduction in oocyst prevalence and intensity due to *w*AlbB. These results are similar to those reported for *P. falciparum* in the stably transinfected line of *An. stephensi* at 27°C^4^. At cooler temperatures we found no effects on prevalence but an increase in oocyst intensity (significant at 24°C). This is in accord with studies on another rodent malaria, *P. berghei*, which showed no impact on prevalence, but enhancement of oocyst intensities in somatically transinfected *An. gambiae* housed at 19°C^39^.

The mechanisms underpinning the diverse transmission blocking phenotypes require further study. There is some evidence that *w*AlbB induces the production of reactive oxygen species (ROS), as well as the upregulation of immune genes like *TEP1* and *LIRM-1*^20^, and that this can inhibit initial *Plasmodium* infection^4^,^21^. How this would lead to enhancement under certain temperatures is unclear but the timing of midgut microbiota proliferation, ROS production, immune gene expression, and parasite kinetics directly following the blood meal might be crucial for parasite blocking at early stages of malaria infection. These different processes are all likely temperature sensitive but need not show identical responses^34^. In addition, the rodent malarias might initiate different responses since ookinetes typically form and migrate through the midgut more rapidly (12–24 hr post-infection) than *P. falciparum* (48 hr)^41^. Other elements of mosquito immune response also differ between rodent and human malaria. The Toll pathway primarily regulates mosquito defense at the oocyst stage against rodent malarias, while the IMD pathway is important for defense against *P. falciparum*^42^,^43^,^44^. Such differences might lead to contrasting patterns of blocking depending on which genes are modulated by *w*AlbB infection and how immune mechanisms are affected by temperature^21^. Variation in *w*AlbB densities within the mosquito could also play a role, although previous research on somatically infected *An. gambiae* suggests there is no direct relationship between oocyst burdens and *w*AlbB densities for either *P. berghei* or *P. falciparum*^21^,^39^.

The impact of *w*AlbB infection on sporozoite development is a completely novel finding. Overall, *w*AlbB reduced the number of sporozoites produced per oocyst across experimental temperatures. This effect counterbalanced the substantial enhancement of oocysts at 24°C leading to no net impact of *w*AlbB on overall infection intensity, while further strengthening blocking at 28°C. The link with oocyst density suggests that the effect could be due in part to direct or indirect resource-mediated competition with sporozoite replication inhibited when *w*AlbB and/or malaria parasite density are high^45^. The presence of *Wolbachia*, combined with host resource demands, could limit the amount or accessibility of resources available for *Plasmodium* development^15^. *Wolbachia* and *Plasmodium* are amino acid heterotrophs and have important and potentially overlapping lipid requirements^46^,^47^,^48^,^49^. Whether *Wolbachia* infection further affects the viability and invasibility of *Plasmodium* sporozoites needs to be further investigated in this system.

In this study we demonstrate complex effects of ambient temperature on the *Wolbachia*-malaria interaction that alter the nature and extent of pathogen blocking. Mean temperatures and daily temperature ranges in natural transmission environments can far exceed those considered here. Based on our data it would be remarkable if the interactions between *Wolbachia*, the mosquito vector and human malaria parasites were unaffected by such variation. Moreover, the impact of *w*AlbB on species such as *P. vivax* or on mixed infections remains unexplored; the potential for enhancement need not be limited to rodent malaria. Further development of this promising control technology requires an improved understanding of how mosquitoes, *Wolbachia* and malaria parasites will interact in diverse transmission settings.

## Methods

### Mosquito rearing, experimental design, and infections

We reared *Anopheles stephensi* (Liston) under standard insectary conditions at 26 ± 0.5°C, 80% humidity, and a 12 h light: 12 h dark photo-period and on a 10% glucose solution diet^32^. *Wolbachia* (*w*AlbB, isolated from *in vitro* cultivation of *Wolbachia pipientis* in an *Ae. albopictus* cell line^50^) was cultured and extracted from *w*AlbB-infected Sua5B *Anopheles* cells and suspended in Schneider's insect cell media as previously described^31^,^51^,^52^. On day three post-emergence, adult female mosquitoes were randomly allocated to one of three treatment groups: 1) unmanipulated, 2) injected with 0.2 μL Sua5B cell lysates and Schneider's insect cell media, or 3) injected with 0.2 μL of *Wolbachia* (5 × 10^6^ *Wolbachia* per mL; 1,000 bacteria per dose). Mosquitoes that were challenged with Sua5B cell lysates or *Wolbachia* received intrathoracic injection into the anepisternal cleft^32^ with a mouth pipette and microcapillary glass needle. Immediately after injection, 1200 mosquitoes from each treatment group (3600 mosquitoes total) were placed into cages (20 × 20 × 20 cm, 80 mosquitoes per cage) and randomly distributed across five Percival incubators set to different experimental temperatures (20°C, 22°C, 24°C, 26°C, and 28°C ± 0.5°C, 80% humidity at a 12 hr light: 12 hr dark photo-period) and three replicates. These experimental temperatures reflect a realistic temperature range for *P. yoelii* transmission, which has a thermal optimum around 24°C for parasite transmission and development^35^,^53^.

To maintain experimental tractability, on day eight post-infection with *Wolbachia*, mosquitoes from each treatment group and replicate that were housed across three experimental temperatures (20°C, 24°C, and 28°C) received a malaria infectious bloodmeal from a mouse (C57 BI/6, >6 weeks old) infected with 10^5^ *Plasmodium yoelii* parasites (clone 17XNL, from the WHO Registry of Standard Malaria Parasites, University of Edinburgh, UK) four days prior. Individuals that did not feed were removed from the cage. Mosquitoes were maintained at 24°C, the thermal optimum for *Plasmodium yoelii* development and replication^35^, for two hours following the 30 min bloodfeed to allow for gamete and zygote formation. After infection, individuals were placed back into their respective temperature treatments and provided with cotton balls moistened with 10% glucose offered *ad libitum*. Throughout the duration of the experiment, we counted and removed dead mosquitoes in each cage daily to quantify the effects of treatment and temperature on daily mosquito mortality.

### Quantifying the effects of temperature on *Wolbachia* dissemination

We destructively sampled 10 mosquitoes per cage across all temperatures on days 8, 12, 16, and 20 post-*Wolbachia* infection. Mosquitoes were killed and immediately frozen at −80°C for future qPCR analyses. DNA was extracted from whole mosquito carcasses using the insect supplement in the 96-well DNeasy Blood and Tissue kit (Qiagen, Inc.) as per the manufacturer's protocol. We performed all qPCR analyses on an ABI Prism 7500 Sequence Detection System (TaqMan) using the Rotor-Gene SYBR Green PCR kit as per the manufacturer's protocol (Qiagen, Inc.) and the following cycler conditions (40 cycles total: activation, 95°C for 5 min; denaturation, 40 cycles run at 95°C for 5 sec each; and annealing and extension, 60°C for 30 sec). *w*AlbB was amplified from mosquito carcasses with modified GF and BR primers which specifically bind to the *wsp* gene^54^. The relative abundance of *w*AlbB strain was determined after normalization to the mosquito single-copy *rpS7* gene^21^,^32^. All qPCRs were completed in triplicate, and the average efficiency of each assay was determined by quantifying the slope of a standard curve of five 1:10 serial dilutions from a positive sample in duplicate. *Wolbachia* densities (presented as a fold-change relative to *rpS7* DNA) were generated using the qGENE software^55^.

### Quantifying the effects of temperature on *Plasmodium* blocking

We compared how oocyst prevalence, intensity, and sporozoite replication varied with *Wolbachia* infection and temperature (20°C, 24°C, and 28°C). To quantify parasite prevalence and intensity, we removed and immediately killed 20 mosquitoes from each treatment and temperature group with chloroform and dissected their midguts in 1× phosphate-buffered saline solution under a standard dissecting scope. Using a compound microscope, we noted whether a midgut was infected or uninfected, and counted the number of *Plasmodium* oocysts that had established in each infected midgut. Midguts were then immediately stored in 95% ethanol for future molecular analyses to assess *Plasmodium* replication and potential sporozoite production. To account for thermal shifts in *Plasmodium* development rates^35^, dissections were staggered to ensure midguts were sampled at approximately the same stage of oocyst development (day 7, 8, and 10 post-malaria infection for 24°C, 28°C, and 20°C, respectively).

Due to inconsistent salivary gland invasion by *P. yoelii* sporozoites, we used sporozoite production per midgut and per oocyst as proxies to estimate transmission potential. To assess sporozoite production, DNA was extracted from individual midguts collected during oocyst dissection using the E.Z.N.A. MicroElute Genomic DNA kit (Omega Bio-Tek, as per the manufacturer's protocol). DNA was eluted in 20 μL of elution buffer, and the number of parasite genomes present in midguts was quantified using a previously developed qPCR assay^56^. Sporozoite production per oocyst was evaluated by dividing the total number of sporozoites per midgut by the number of oocysts quantified for each midgut.

### Statistical analyses

All statistical analyses for these experiments were run in IBM SPSS Statistics 21.0 (IBM Corporation). Full factorial models from generalized linear model (GZLM) analysis were reduced through backward elimination of non-significant interactions. We assessed goodness of fit of the final models through model deviance, log likelihood values, and model residuals. Covariates included in GZLMs were centred on their grand mean, and adjusted Bonferroni post-hoc tests were used to identify significant pair-wise comparisons. For all dependent variables analyzed, we included the following factors in our model analysis: temperature (*Wolbachia* densities and mosquito survival, 20°C, 22°C, 24°C, 26°C, and 28°C; *Plasmodium* blocking, 20°C, 24°C, and 28°C), treatment (unmanipulated, Sua5B, and *w*AlbB), and replicate.

We used a GZLM assuming a normal distribution to determine how *Wolbachia* density was affected by our experimental treatment groups. In addition to the fixed factors mentioned above, we included sampling date post-*Wolbachia* infection in our model analysis (day 8, 12, 16, and 20 PI). We used a Poisson GZLM to quantify the effects of experimental treatment on the average number of infected mosquitoes and a negative binomial (log link function) GZLM to quantify the effects of experimental treatment on oocyst intensity. Because of differences in parasite replication rates due to temperature, we ran a series of GZLM analyses (assuming either a normal distribution on transformed sporozoite data or a gamma distribution on untransformed data) on the total number of *Plasmodium* sporozoites per midgut and per oocyst independently for each temperature. Because midguts with more oocysts likely produce more sporozoites, we also included oocyst intensity as a covariate in both analyses. Finally, we used Kaplan-Meier survival analyses to generate cumulative daily survival estimates to determine the effects of temperature and treatment on daily mosquito survival throughout the course of the experiment.

## Author Contributions

C.C.M. contributed to conceiving and designing experiments, running the experiments, analyzing data associated with the experiments, and in writing the manuscript. S.B. contributed to designing and running the experiments. G.L.H. contributed to designing the experiment, provided tools and techniques for the experiment, and in writing the manuscript. J.L.R. contributed by providing resources for the experiment and in writing the manuscript. M.B.T. contributed to conceiving the experiments, providing resources for the experiments, and in writing the manuscript.

## Supplementary Material

Supplementary Information Supplementary Information

## Figures and Tables

**Table 1:** Generalized linear model analysis of the effect of experimental treatment on wAlbB density, oocyst prevalence, and oocyst intensity

	*wAlbB density* (n = 294)			*oocyst prevalence* (n = 60)			*oocyst intensity* (n = 538)		
factors	Wald *X*^2^	d.f.	*p*	Wald *X*^2^	d.f.	*p*	Wald *X*^2^	d.f.	*p*
*intercept*	**232.07**	**1**	**<0.0001**	**1083.68**	**1**	**<0.0001**	**3268.79**	**1**	**<0.0001**
*temperature*	**130.58**	**4**	**<0.0001**	**74.49**	**4**	**<0.0001**	**760.88**	**2**	**<0.0001**
*treatment*	-	-	-	0.96	3	0.812	5.07	3	0.167
*replicate*	2.45	2	0.294	2.85	2	0.241	3.71	2	0.157
*days post-wolbachia infection*	**138.07**	**3**	**<0.0001**	-	-	-	-	-	-
*temperature x treatment*	-	-	-	-	-	-	**105.69**	**6**	**<0.0001**
*days post-wolbachia infection x temperature*	**84.29**	**12**	**<0.0001**	-	-	-	-	-	-

Omnibus tests confirmed that each fitted model was significantly different from its null model (*wAlbB density*: likelihood ratio *X*^2^*_1, 21_* = 227.42, p < 0.0001; *oocyst prevalence*: likelihood ratio *X*^2^*_1, 10_* = 44.55, p < 0.0001; *oocyst intensity*: likelihood ratio *X*^2^*_1, 10_* = 553.48, p < 0.0001). Goodness of fit was assessed by evaluating potential overdispersion through model deviance scores and model residuals (*wAlbB density*: normal distribution, deviance value/d.f. = 3.62; *oocyst prevalence*: Poisson distribution, deviance value/d.f. = 1.12; *oocyst intensity*: negative binomial distribution, deviance value/d.f. = 3.35).

**Table 2:** Generalized linear model analysis of the effect of treatment on the number of sporozoites produced per oocyst and midgut analyzed independently for each experimental temperature

	*20°C* (n = 116)			*24°C* (n = 118)			*28°C* (n = 42)		
factors	Wald *X^2^*	d.f.	*p*	Wald *X^2^*	d.f.	*p*	Wald *X^2^*	d.f.	*p*
***sporozoites/oocyst***									
*intercept*	**2581.88**	**1**	**<0.0001**	**5628.47**	**1**	**<0.0001**	**420.03**	**1**	**<0.0001**
*treatment*	**9.98**	**2**	**0.007**	**25.86**	**2**	**<0.0001**	**11.45**	**2**	**0.003**
*replicate*	0.78	2	0.678	**11.13**	**2**	**0.004**	2.39	2	0.303
*centred oocyst intensity*	**9.79**	**1**	**0.002**	1.53	1	0.216	**12.76**	**1**	**<0.0001**
*treatment x centred oocyst intensity*	-	-	-	-	-	-	**11.96**	**2**	**0.003**
***sporozoites/midgut***									
*intercept*	**11912.35**	**1**	**<0.0001**	**8610.63**	**1**	**<0.0001**	**1581.66**	**1**	**<0.0001**
*treatment*	2.89	2	0.236	**28.50**	**2**	**<0.0001**	**15.14**	**2**	**0.001**
*replicate*	0.55	2	0.759	**10.45**	**2**	**0.005**	**7.70**	**2**	**0.021**
*centred oocyst intensity*	**97.52**	**1**	**<0.0001**	**1497.95**	**1**	**<0.0001**	0.15	1	0.699
*treatment x centred oocyst intensity*	**6.64**	**2**	**0.036**	-	-	-	**20.96**	**2**	**<0.0001**

Omnibus tests confirmed that each fitted model was significantly different from its null model (*sporozoites/oocyst*: *20°C* - likelihood ratio *X*^2^*_1, 7_* = 122.09, p < 0.0001; *24°C* - likelihood ratio *X*^2^*_1, 5_* = 309.95, p < 0.0001; *28°C* - likelihood ratio *X*^2^*_1, 7_* = 25.42, p < 0.0001; *sporozoites/midgut*: *20°C* - likelihood ratio *X*^2^*_1, 7_* = 122.09, p < 0.0001; *24°C* - likelihood ratio *X*^2^*_1,7_* = 315.16, p < 0.0001; *28°C* - likelihood ratio *X*^2^*_1, 7_* = 39.98, p < 0.0001). Goodness of fit was assessed by evaluating potential overdispersion through model deviance scores and model residuals. *Sporozoite/oocyst* data were transformed and fit to normal distributions (*20°C* - deviance value/d.f. = 1.57; *24°C* - deviance value/d.f. = 4.92; *28°C* - deviance value/d.f. = 1.13). *Sporozoite/midgut: 20°C* - gamma distribution, deviance value/d.f. = 1.57; *24°C* - transformed data fit to a normal distribution, deviance value/d.f. = 4.92; *28°C* - gamma distribution, deviance value/d.f. = 1.13.
