# Supplementary material for: Temperature alters Plasmodium blocking by Wolbachia
Source: Sci Rep. 2014 Feb 3;4:3932. doi: 10.1038/srep03932 (PMC3909897; doi:10.1038/srep03932)

Supplementary Information

Title: Temperature alters *Plasmodium* blocking by *Wolbachia*

C. C. Murdock[1], Blanford, S.[1], Grant L. Hughes[1], Jason L. Rasgon[1], and Matthew B. Thomas[1]

[1]Center for Infectious Disease Dynamics and Department of Entomology, Pennsylvania State University, University Park, PA, USA.

Table S1

Phenotype	System	Temperature	Result	Reference
Wolbachia density	*Nasonia vitripennis* (wasp)	18ºC, 25ºC, 30ºC	densities were significantly lower at the cooler and warmer temperatures, relative to wasps held at 25ºC	1
	Exorista sorbillans (Uzifly)	26ºC and 33ºC	*Wolbachia*was eliminated at 33ºC relative to *Wolbachia*-infected females at 26ºC	2
	Tetranychus urticae (spider mite)	19ºC, 22ºC, 25ºC, 28ºC, and 31ºC	*Wolbachia* density was highest in both males and females at 25ºC in the ZJ mite strain, and was highest in YC female mites at 25ºC and in YC males at 28ºC relative to mites reared at cooler and warmer temperatures.	3
	Leptopilina heterotoma (wasp)	20ºC and 26ºC	A7 females reared at 20ºC experienced significantly lower *Wolbachia* density, while *Wolbachia* densities in SF4 females remained unaffected by temperature.	4, 5
	Aedes albopictus (Asian tiger mosquito)	25ºC and 37ºC	Elevated temperature significantly decreased *Wolbachia* density in all stages in both males and females	6
	Aphytis melinus (wasp)	24ºC, 25ºC, 27.5ºC, 30ºC, and 32.5ºC	*Wolbachia* densities decreased in wasps reared at warmer temperatures (30ºC and 32.5ºC) relative to cooler temperatures.	7
Cytoplasmic incompatability	*Nasonia vitripennis* (wasp)	18ºC, 25ºC, 30ºC	proportion of crosses expressing complete CI was significantly greater in wasps reared at extreme temperatures (18ºC and 30ºC) relative to those reared at 25ºC	1
	Tetranychus urticae (spider mite)	19ºC, 22ºC, 25ºC, 28ºC, and 31ºC	CI was highest in ZJ mites reared at 28ºC relative to ZJ mites reared at warmer or cooler temperatures.	3
	Tetranychus urticae (spider mite)	23ºC and 32ºC	CI occurred between *Wolbachia*-infected males and previously infected females due to a loss of *Wolbachia* in mites reared at warmer temperatures.	8
	Porcellionides pruinosus (wood lice)	20ºC and 30ºC	Variation in *Wolbachia* densities may influence sex determination in wood lice, with highly female-biased broods and male-biased broods occurring at 20ºC and 30ºC, respectively, in three different field populations.	9
Vertical transmission	*Drosophila simulans* (fruit fly)	19ºC and 25ºC	exposure to 25ºC reduced the density of *Wolbachia* in embryos compared with flies exposed to 19ºC	10
	Drosophila bifasciata (fruit fly)	18ºC, 21ºC, 23.5ºC, 25ºC	exposure to 25ºC reduced the transmission efficiency of *Wolbachia* and male killing through reductions in *Wolbachia* densities	11
	Loposcelis tricolor (psocids)	28ºC and 33ºC	*Wolbachia* infection was lost after six generations when reared at 33ºC	12
	Tetranychus urticae (spider mite)	23ºC and 32ºC	*Wolbachia* infection was reduced after four generation and lost after six generations in mites reared at the warmer temperature	8

Fitness	*Exorista sorbillans* (Uzifly)	26ºC and 33ºC	*Wolbachia*was eliminated at 33ºC, and female fecundity and egg hatchability decreased significantly relative to *Wolbachia*-infected females at 26ºC. Fecundity and hatchability also varied seasonally with temperature.	2
	Drosophila melanogaster (fruit fly)	19ºC and 25ºC	*Wolbachia*-infected flies had significantly shorter lifespans at 25ºC only, potentially due to lower *Wolbachia* replication at 19ºC.	13

Literature Cited

1 Bordenstein SR, Bordenstein SR (2011) Temperature affects the tripartite interactions between bacteriophage WO, *Wolbachia*, and cytoplasmic incompatability. Plos One 6, doi:e29106 10.1371/journal.pone.0029106.

2 Guruprasad NM, Mouton L, Puttaraju HP (2011) Effect of *Wolbachia* infection and temperature variations on the fecundity of the Uzifly *Exorista sorbillans* (Diptera: Tachinidae). Symbiosis 54: 151-158, doi:10.1007/s13199-011-0138-y.

3 Lu M-H, Zhang K-J, Hong X-Y (2012) Tripartite associations among bacteriophage WO, *Wolbachia*, and host affected by temperature and age in *Tetranychus urticae*. Exp. Appl. Acarol. 58: 207-220, doi:10.1007/s10493-012-9578-1.

4 Mouton L, Henri H, Bouletreau M, Vavre F (2006) Effect of temperature on *Wolbachia* density and impact on cytoplasmic incompatibility. Parasitology 132: 49-56, doi:10.1017/s0031182005008723.

5 Mouton L, Henri H, Charif D, Bouletrea M, Vavre F (2007) Interaction between host genotype and environmental conditions affects bacterial density in *Wolbachia* symbiosis. Biology Letters 3: 210-213, doi:10.1098/rsbl.2006.0590.

6 Wiwatanaratanabutr I, Kittayapong P (2009) Effects of crowding and temperature on *Wolbachia* infection density among life cycle stages of *Aedes albopictus*. J. Invertebr. Pathol. 102: 220-224, doi:10.1016/j.jip.2009.08.009.

7 Vasquez CJ, Stouthamer R, Jeong G, Morse JG (2011) Discovery of a CI-inducing *Wolbachia* and its associated fitness costs in the biological control agent *Aphytis melinus* DeBach (Hymenoptera: Aphelinidae). Biological Control 58: 192-198, doi:10.1016/j.biocontrol.2011.06.006.

8 Van Opijnen T, Breeuwer JAJ (1999) High temperatures eliminate *Wolbachia*, a cytoplasmic incompatibility inducing endosymbiont, from the two-spotted spider mite. Exp. Appl. Acarol. 23: 871-881, doi:10.1023/a:1006363604916.

9 Rigaud T, Antoine D, Marcade I, Juchault P (1997) The effect of temperature on sex ratio in the isopod *Porcellionides pruinosus*: Environmental sex determination or a by-product of cytoplasmic sex determination? Evolutionary Ecology 11: 205-215, doi:10.1023/a:1018404000804.

10 Clancy DJ, Hoffmann AA (1998) Environmental effects on cytoplasmic incompatibility and bacterial load in *Wolbachia*-infected *Drosophila simulans*. Entomologia Experimentalis et Applicata 86: 13-24, doi:10.1046/j.1570-7458.1998.00261.x.

11 Hurst GDD, Jiggins FM, Robinson SJW (2001) What causes inefficient transmission of male-killing *Wolbachia* in *Drosophila*? Heredity 87: 220-226, doi:10.1046/j.1365-2540.2001.00917.x.

12 Jia FX, Yang MS, Yang WJ, Wang JJ (2009) Influence of continuous high temperature conditions on *Wolbachia* infection frequency and fitness of *Liposcelis tricolor* (Psocoptera: Liposcelididae). Environmental Entomology 38: 1365-1372.

13 Reynolds KT, Thomson LJ, Hoffmann AA (2003) The effects of host age, host nuclear background and temperature on phenotypic effects of the virulent *Wolbachia* strain popcorn in *Drosophila melanogaster*. Genetics 164: 1027-1034.

Text S2

Survival Analyses

Text S2 Figure 1 The effects of temperature and treatment on cumulative mosquito survival. **A**There were no significant effects of experimental temperature on cumulative mosquito survival. **B** Treatment did affect mosquito mortality, with significant declines in cumulative mosquito survival in Sua5B lysate and wAlbB injected mosquitoes within the first two days post-injection. However, this mortality is most likely due to the intrathoracic injection technique because the slopes of the survival curves are equivalent across all treatments three-four days following injection.

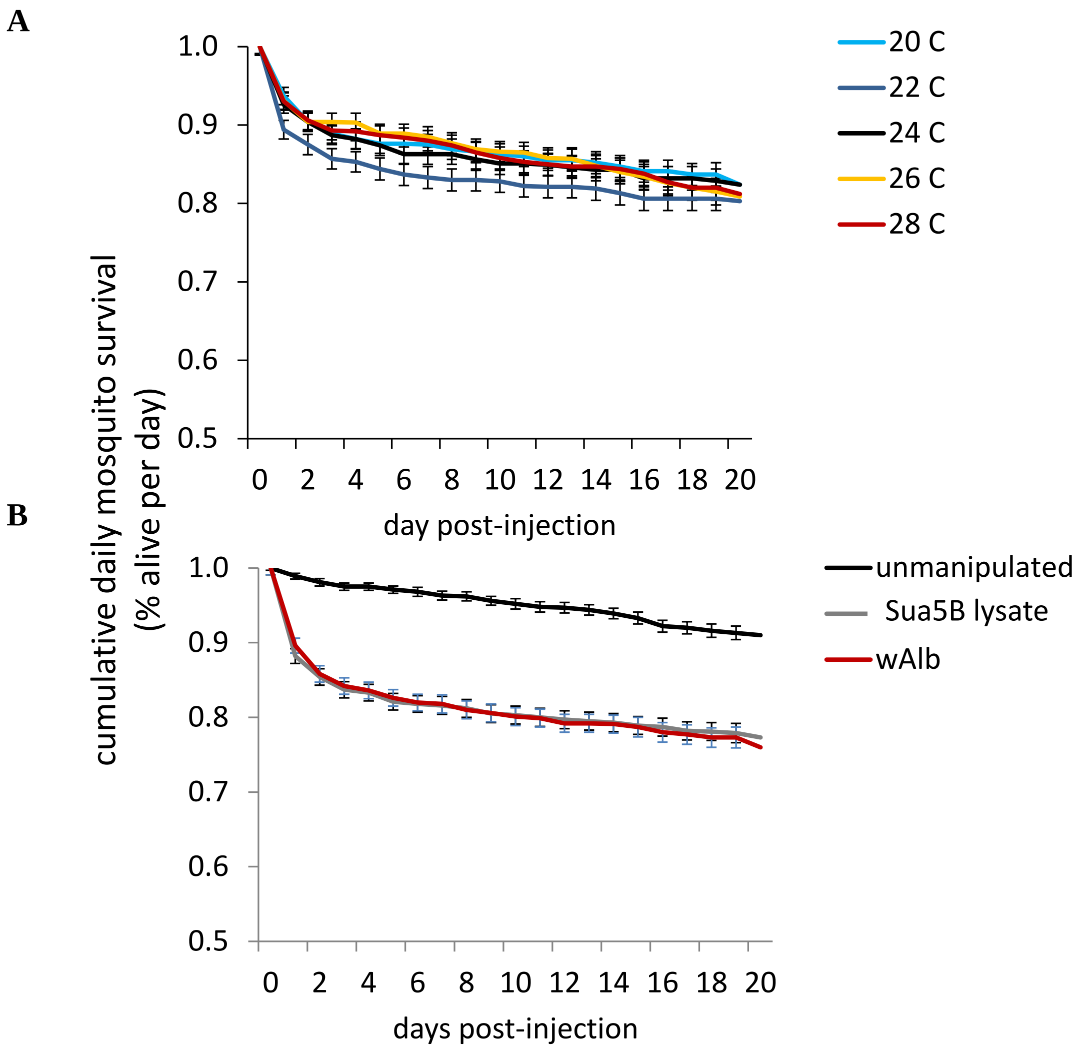

Sporozoite Production per Midgut at 24ºC

To investigate the discrepancy between the unadjusted means represented in Fig 2d and the model predicted estimates for the mean sporozoite production for each treatment group at 24ºC, we conducted a series of additional analyses. When the covariate oocyst intensity was removed from the generalized linear model analysis (GZLM), the model no longer predicted a significant treatment effect. This is because much of the variation explained by the model can be attributed to the positive effect of oocyst intensity on total sporozoite production (model with covariate included, AICC 534.7 vs. model without covariate, AICC 841.2). To further examine the effect of treatment on the variation that is not explained by the covariate oocyst intensity, we ran a linear regression with oocyst intensity as the independent variable and sporozoite production per midgut as the response variable (R^2 = 0.927, $F_{1,16}$ = 1475.34, n = 118, p < 0.0001, slope = 7845.73; Text S2 Fig 2).

Text S2 Figure 2 There is a strong positive relationship between the number of oocysts and the total number of sporozoites a malaria infected midgut produces.

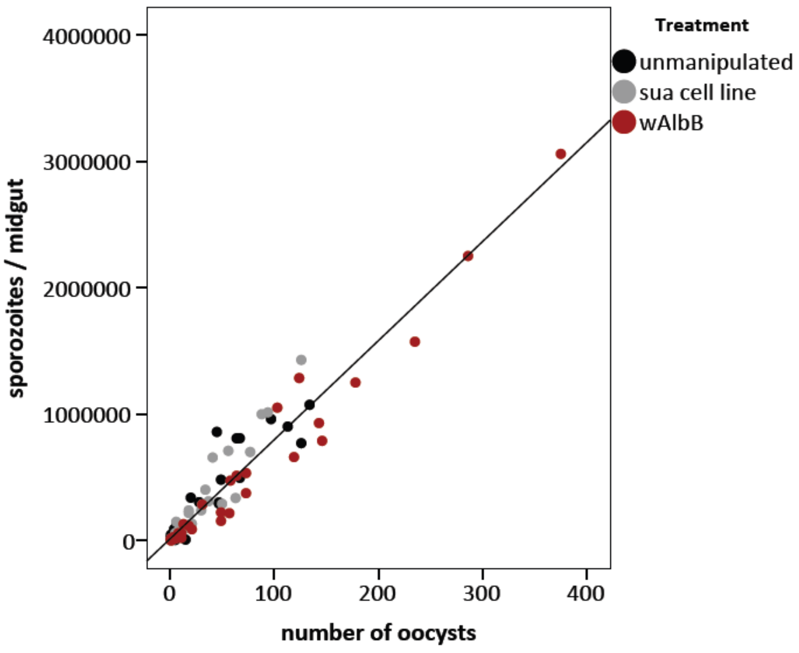

We then ran a GZLM with treatment and replicate as factors on the residuals (unexplained variation) from the regression model. Treatment significantly predicted the remaining unexplained variation (Wald $X^2_{1,2}$ = 15.03, p = 0.001), with wAlbB infection significantly reducing sporozoite production per midgut relative to the control

treatment groups (unmanipulated vs. wAlbB, $p = 0.008$; Sua5B cell lysate vs. wAlbB, $p = 0.003$). Thus, wAlbB infected mosquitoes were more likely to fall below the regression line in regards to sporozoite production while the control groups were more likely to be above (represented as 0 in Text S2 Fig 3).

Text S2 Figure 3 Infection with wAlbB significantly reduces the total number of sporozoites produced per midgut relative to the control treatment groups after the positive relationship between oocyst intensity and sporozoite production has been accounted for. These results demonstrate why the initial GZLM analysis predicted a significant effect of treatment despite the apparently non-significant differences across treatment groups illustrated by the unadjusted means at 24°C (Fig 2).

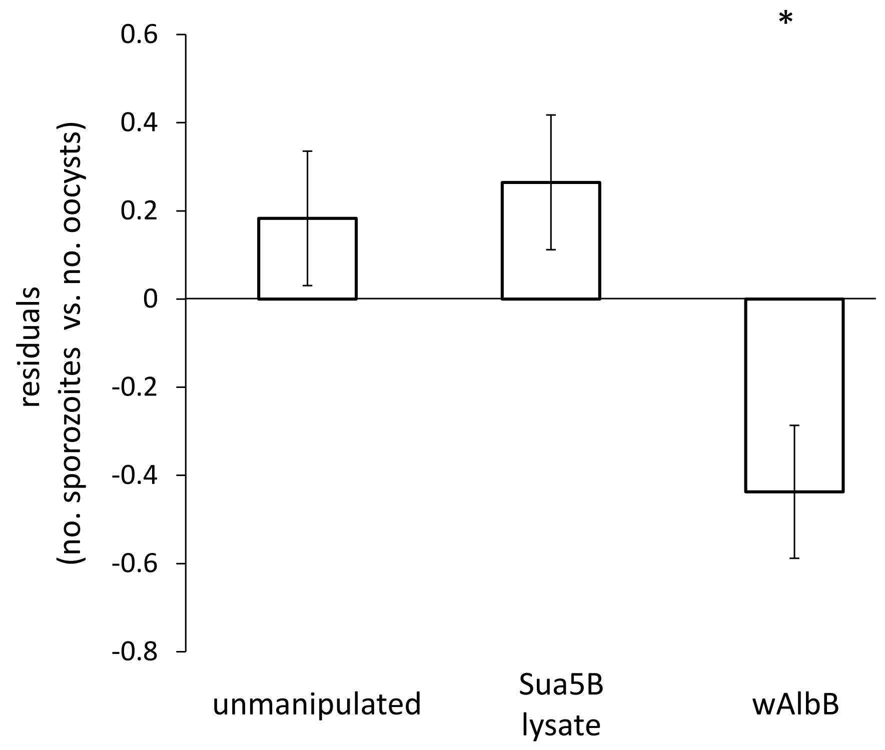

Supplement: Supplementary Information [file srep03932-s1.pdf]
